# Developing a critical realist informed framework to explain how the human rights and social determinants of health relationship works

**DOI:** 10.1186/s12889-019-7760-7

**Published:** 2019-11-27

**Authors:** Fiona Haigh, Lynn Kemp, Patricia Bazeley, Neil Haigh

**Affiliations:** 1 Centre for Health Equity Training, Research & Evaluation (CHETRE), UNSW Sydney, Sydney, Australia; 2 Ingham Institute, Sydney, Australia; 3 Translational Research and Social Innovation Unit (TReSI), Western Sydney University, Sydney, Australia; 4 EdQuest, Hamilton, New Zealand

**Keywords:** Human rights, Social determinants of health, Critical realism, Health equity, Explanatory model, Paradigm

## Abstract

**Background:**

That there is a relationship between human rights and health is well established and frequently discussed. However, actions intended to take account of the relationship between human rights and social determinants of health have often been limited by lack of clarity and ambiguity concerning how these rights and determinants may interact and affect each other. It is difficult to know what to do when you do not understand how things work. As our own understanding of this consideration is founded on perspectives provided by the critical realist paradigm, we present an account of and commentary on our application of these perspectives in an investigation of this relationship.

**Findings:**

We define the concept of paradigm and review critical realism and related implications for construction of knowledge concerning this relationship. Those implications include the need to theorise possible entities involved in the relationship together with their distinctive properties and consequential power to affect one another through exercise of their respective mechanisms (ways of working). This theorising work enabled us identify a complex, multi-layered assembly of entities involved in the relationship and some of the array of causal mechanisms that may be in play. These are presented in a summary framework.

**Conclusion:**

Researchers’ views about the nature of knowledge and its construction inevitably influence their research aims, approaches and outcomes. We demonstrate that by attending to these views, which are founded in their paradigm positioning, researchers can make more progress in understanding the relationship between human rights and the social determinants of health, in particular when engaged in theorizing work. The same approaches could be drawn on when other significant relationships in health environments are investigated.

## Context and case

Global initiatives such as the WHO Commission on Social Determinants of Health, the 2011 Rio Declaration, and 2015 Sustainable Development Goals, identify human rights as key to addressing inequities in social determinants of health. Correspondingly, there have also been calls from human rights monitoring bodies – including the United Nations (UN) Commission on Human Rights, the UN Committee on the Rights of the Child, the UN Committee on Economic, Social and Cultural Rights and the UN Special Rapporteur on the right to health [1–3] - for the development of health impact assessment tools and approaches that can provide insights into ways government actions affect the right to health. However, action specifically based in a human rights approach to identifying and addressing social determinants of health has been limited and these major global initiatives have been critiqued. While acknowledging the role of rights, few initiatives have explicitly attempted to incorporate rights into actions and priorities [4–8]. Chapman describes how

reticence to recognize the shared agenda and potential contribution of the human rights paradigm is particularly surprising in view of the Commission secretariat’s recommendation that the CSDH adopt a rights-based approach as an appropriate conceptual framework to advance towards health equity through action on the social determinants of health [5]

However, we think that this situation is not unsurprising as there is currently a lack of underpinning understanding of how human rights (HRs) and social determinants of health (SDOH) interact and affect each other: how the relationship can ‘work’. Further, there are differing conceptualisations of the determinants of health used in human rights and public health that have important implications for how relationships between SDOH and health rights are understood [4, 7]. For example, human rights conceptualisations of social determinants of health often fail to take into account how determinants interact with each other and also to consider the structural determinants of health [5]. Current human rights interpretations of the right to the highest attainable standard of health and healthcare and health determinants contained in reports from human rights bodies may miss important causes due to human rights narrower conceptualisation of determinants of health. Conceptual models used to understand and describe how the SDOH shape people’s lives are often limited to a narrow range of causal pathways that reflect particular disciplinary perspectives [9–11].

We also propose that these apparent disciplinary differences may reflect, in turn, more fundamental differences and variations in points of view about reality, the nature of knowledge that we attempt to construct about what we construe to be real and how we should go about constructing and evaluating knowledge: different ‘paradigms’ may be in play. From this perspective, we believe that attempts to develop knowledge about particular phenomena require explicit attention by researchers to their ‘paradigm positioning’. As Carter and Little [12] observe, it is impossible to create knowledge “without at least tacit assumptions about what knowledge is and how it is constructed”. Conversely, those who read accounts of such attempts need to take into account the paradigm position of the researchers.

To clarify and illustrate the implications of this stance, we define the notion of a paradigm, outline the key tenets of our own paradigm position – critical realism, and then describe in detail how we applied these tenets to develop theory about the relationship between human rights and the social determinants of health.

To demonstrate key points, we use a case study of the Vermont Right to Health Care Campaign [13]. Vermont is a small state in the northeast of the USA with a population of just over 600,000. The United States does not have a Universal Health Care (UHC) system. Healthcare is paid for through a mix of private insurance and government funded health insurance schemes for particular population groups. In 2008, the Vermont Workers’ Center (VWC) began a “Health Care is a Human Right” campaign. The campaign adopted human rights principles to guide all its work. The VWC developed a staged approach which first focussed on building power through activating Vermonters, then directly targeting the legislature. We applied a CR explanatory framework to explain how a human rights-based approach can work to influence access to health care. Details of the case study are described in a separate publication [13]. In conjunction with this case study, we provide a reflective critique on our use of a CR-based theorizing methodology.

## The concept of a research paradigm

*The matter with human beans is that they is absolutely refusing to believe in anything unless they is actually seeing it right in front of their own schnozzles* The BFG

We understand a paradigm to constitute four categories of interrelated views that underpin our conceptions of knowledge and knowing: *ontology* – one’s understanding of the nature of reality and what can be known about that reality; *epistemology* – understanding of the nature of knowledge, the ‘getting to know’ process, the relationship between the person who seeks to know and the knowledge they construct, and the criteria for making claims about knowledge; *methodology* – approach to the construction of knowledge; and *axiology* – the influence of values on knowledge that is acquired and how it is acquired. A coherent set of views in relation to these four considerations constitutes a paradigm position.

As previously noted, different disciplines and subject matter fields have developed traditions in relation to these views. For example, medical sciences have tended to adopt a positivist or post-positivist paradigm, based on the view that what is real, and therefore knowable, is what can be observed ‘out there’ and measured. This perspective is also apparent in some conceptions of human rights as legal rules found within treaties [14]. In contrast, social sciences often adopt a social constructivist paradigm which rests on the view that what is real is what our individual minds ‘make’ real to us; reality is a construction – by and of the mind. And, the knowledge that we construct about these in-the-mind realities is influenced by the social relationships in which we are embedded. From this perspective, “there exist multiple, socially constructed realities ungoverned by natural laws, causal or otherwise” [15]. The relationship between different fields and paradigm positions is more nuanced than presented here and within specific fields there exist a mix paradigm perspectives [16, 17] but for the purposes of this paper the main point is that differing ontological and epistemological positions have implications for the questions researchers seek to answer, the methodologies they employ, the data they gather - and the ways in which data are gathered, analysed and interpreted. For example, while social constructionists are more likely than positivists to be interested in investigating qualitative differences in the meanings people give to experiences, positivists are more likely to be interested in identifying stable relationships between things and substantiating these relationships using generalisable quantitative data. Differences in paradigm positioning might also be linked to different social groups or cultures. For example, in New Zealand researchers give explicit consideration to Maori ontology and epistemology [18] and Maori specific research methodology (Kaupapa Maori). Some researchers, especially those employing mixed methods, adopt a pragmatic paradigm position in which their view of reality is based on and tested through experience. They choose methods, therefore, based on their experience of what works best for answering their research questions. While some researchers have an explicit awareness of their paradigm position and communicate it in research publications, others have an implicit position only.

## Critical realism research paradigm – key features and relevance to human rights and social determinants of health

Critical realism (CR) is a relatively new paradigm position. It represents a combination of views that contrast with those associated with traditional positivist and interpretivist positions [19–21]. An increasing number of public health, and to a lesser extent human rights, scholars are adopting a CR position [e.g] [9, 22–25]. There is also now a large body work in the area of realist evaluation which is informed by a critical realist research paradigm [26], including examples in this journal [e.g] [27–29].

In the following sections we briefly elaborate on the key features of the critical realist research paradigm.

1. According to CR, there is a reality that exists independent of our thoughts about it, and while observing may make us more confident about what exists, existence itself is not dependent on observation [19]. An example of this is that people have the right to health even when they are not aware of it. While we can acquire or construct knowledge about reality, that knowledge can be fallible, or mistaken.
2. Reality is stratified into three domains: empirical, actual and real. The real domain consists of entities or structures which have properties that give them the power to activate mechanisms that can affect other structures (i.e. causal mechanisms); the actual domain consists of events and their effects that have been caused by the activation of causal mechanisms; and the empirical domain represents actual events-effects that can be, or have been, observed or experienced. For example, human rights may be observable at the empirical level through asking people about their beliefs and attitudes towards human rights. The actual level consists of what happens when people’s rights to the determinants of health such as education, housing, health care, freedom from discrimination are fulfilled or neglected. These events-effects can only be explained with reference to the real level, where unseen causal powers associated with such entities as class, gender, and capitalism are triggered.
3. The world is made up of entities that have properties that endow them with powers and liabilities. Events happen when the powers of one or more entities are activated. Because of the stratified nature of reality, entities can be invisible or visible. This means they can include non-physical things such as ideas, theories, concepts or institutions, as well as physical entities such as cigarettes or guns. In the social world, entities are often invisible (e.g. human rights, discrimination, capitalism). These invisible entities are not observable at the empirical level, but the effects of their activated powers/mechanisms may be observable (e.g. health outcomes, access to health services, health service costs, measured inequalities). A CR approach also understands absence of entities as being causally efficacious. Critical realism provides a critique of ‘ontological monovalence’, which is the idea that only things that are present exist [21, 30]. Just as when lack of rain causes a drought, or in the case of Vermont, lack of access to health care causes unmet health needs or lack of respect for rights causes suffering, rights are often most causally powerful and important when they are absent. Activation, which involves the exercise of particular mechanisms, is contingent on other entities and their mechanisms (context).
4. Knowledge is transitive– our understanding of a phenomenon can change. While entities exist independent of our ability to perceive and conceive that they exist, we do use our minds to construct knowledge about them. As the construction of knowledge can never be infallible – sometimes we construct misconceptions or mistaken theories – our knowledge of the world is transitive. It is open to challenge and change. This CR epistemological perspective means that we recognize that theory that we have developed about human rights and health may in time be extended, modified or rejected, notwithstanding our attempt to ensure its trustworthiness and practical adequacy. A theory is not intransitive, as reality is.
5. The social world is a layered, complex and open system. Within this system, multiple entities are present, the types of entities are wide ranging, each entity may subsume other entities or be subsumed within other entities, and a vast array of these entities’ mechanisms may be activated and in play moment by moment. For example, within the Vermont case study, entities that were attended to included organizations such as the Vermont Workers Centre, people such as political representatives, policies such as Health Care Policy, plans including those of the VWC campaign, goals such as improving access to health services, methods and tools such as letter writing and human rights assessment of proposals. Some people had multiple roles (e.g. doctor, campaigner, parent). As each entity had properties that endowed it with mechanisms which could enable, constrain or block the mechanisms of other entities, the actual interactions between entities and their effects were extremely complex. The exercise of mechanisms was often contingent on the mechanisms of another entity being activated. For example, the Vermont Workers Centre had its latent causal powers-mechanisms (e.g. to empower, to inform) activated when a group of people decided to exercise their power to ‘campaign for universal health care’. And, the exercise of some mechanisms was a manifestation of personal power to act (i.e. the exercise of agency by a Vermonter to write a letter) or the power of social structures over personal action (e.g. the activation of compliance mechanisms associated with the rules of accessing the Vermont Legislature). It was evident that causal power could shift between agency and structure. The exercise of some mechanisms (e.g. informing mechanisms of conducting human rights assessments of new proposals) lead to changes in the properties of entities (e.g. Vermont citizens gained knowledge of rights and corresponding state duties) and, in turn, power to exercise new mechanisms (e.g. to claim rights through a right to health rights campaign). They also lead to the emergence of new entities (e.g. new legislative proposals). In this sense, a social system is always open to and characterized by change. This contrasts with a system in which law-like regularities can be identified (e.g. signing human rights treaties invariably leads to decreases in human rights violations). In an open system, such relationships are context dependent [31, 32].

### Critical Realist methodology

From a CR perspective, the primary purpose of research, and therefore of the application of a methodology, is the theorizing of explanations for ‘tendencies’ in phenomena that have been observed or experienced (e.g. events, effects). These explanations focus on the mechanisms of entities that can generate events – as well as the properties of entities that empower them with such mechanisms. Bhaskar describes how “This is the arduous task of science: the production of the knowledge of those enduring and continually active mechanisms of nature that produce the phenomena of our world” (Bhaskar, 1975, p.47).

Tendencies may include recurrent relationships between phenomena, variability in such relationships or the absence of a relationship – and complexity is likely to characterize the interactions between entities and their associated mechanisms. Critical realists are pragmatic in their approach to methodology and methods. Because of the layered nature of reality, multiple disciplines and methodological approaches may be needed to understand the multilevel relationships between human rights and social determinant of health. Research design should be ‘practically adequate’: that is,‘fit for purpose’ [30]. This allows space for the members of different disciplines to work together to understand a topic such as human rights and the social determinants of health.

### Critical Realist axiology

Emancipatory objectives form part of a critical realist research agenda. Danermark points out that “A critical science often takes its starting point in notions that improvement of society is possible” [20].

The implication of this emancipatory worldview is that when phenomena are under investigation it may be possible to identify how these features may be influenced (e.g. properties, and therefore mechanisms, changed) in order to ameliorate harmful effects or to enhance beneficial effects. Thus, CR research has an inherent focus on ‘what to do’ to improve people’s human rights situation.

## Critical realism, the social determinants of health and human rights

In the following sections, we describe how we drew on critical realist perspectives to develop theory about the relationship between human rights and social determinants of health. In doing so, we focus on two processes; structural analysis of human rights and social determinants of health and identifying causal relationships between social determinants of health and human rights. A framework summarizing the outcomes of these analysis and theorising processes is presented.

The general case for attending to paradigm position when undertaking such research is also made.

In order to develop explanatory theory, concerning the relationship between human rights and the social determinants of health, the entities themselves need to be described. What are human rights? What are social determinants of health? Each of these entities has a structure, a set of properties or attributes that differentiate it from other entities. In turn, those properties give the entity the power to activate or exercise mechanisms that can cause effects. These effects may, in turn, involve changes to the properties of an entity and, therefore its potential mechanisms. Description of these entities, from both perspectives (cause and effect), involves structural analysis.

Human rights attributes include the following: rights are *norms*; rights exist within *relationships* between claim holders and duty bearers; rights have core *principles* that provide a *framework* for application; rights have *substantive* and *procedural* elements. These various properties may be further differentiated and described. For examples norms may be universal/community specific, clear/unclear, accepted/contested, non/conflicting. The specifics of properties determine whether and what mechanisms can be activated. In this instance, the mechanisms may include informing, guiding, persuading, preventing and enforcing. These mechanisms are latent because their activation is contingent on the mechanisms of another entity being activated (e.g. someone reads and thinks about the norm). Such contingent relationships are common in social environments. For example, the exercise of mechanisms associated with human rights norms can change the capacity of a community to hold duty bearers accountable for impacts on health and health rights. However, the capacity of rights holders to claim rights may also be contingent on the exercise of the mechanisms of education programs that are intended to facilitate learning about rights and ways of claiming rights (e.g. in Vermont, information derived from a human rights analysis was presented to Vermonters to inform them about how policy changes impacted on human rights obligations).

Social determinants of health are entities that can cause health-related effects on individuals and communities and that have the following general properties: they exist within the social environment, they result from decisions about how societies should be organised and ‘work’ (e.g. social norms, policies, practices, economic arrangements, politics, education) and they may change over time and vary across social groups and contexts. Again, the properties and associated mechanisms of specific entities (e.g. a health policy, housing policy, an education programme) can be elaborated and delineated with much greater precision using CR ontological perspectives and analysis processes. Questions that can help identify the properties of entities include:

- What does the existence of this object/practice presuppose? What are its preconditions?
- Can/could object A exist without B? If so, what else must be present?
- What is it about this object, that enables it to do certain things (there may be several mechanisms at work and we need to seek ways to distinguish their respective efforts)?
- What cannot be removed without making the object cease to exist in its present form?
  [20, 30].

When making a structural analysis of entities, it should not be assumed that entities that share the same name (e.g. disadvantaged community, race, gender, sexuality, disability, and ethnicity) have similar properties and consequential powers. This needs to be taken into account when the applicability of evidence from other research involving similar entities is considered. To what extent do they (e.g. affected communities) have common properties and therefore powers? Are the findings from other research relevant given contrastive properties and powers?

A further caveat concerns the attention that is given to what can be observed (the empirical domain). Critical realists contest the notion that what can be observed and measured is the thing itself [31]. This view, that Bhaskar calls the epistemic fallacy, reduces statements about the world (ontology) to statements about our knowledge of the world (epistemology) [21]. We see epistemic fallacy in some existing approaches to the right to health, that tend to focus on identifying changes to indicators. Indicators are used as proxies for human rights (e.g. ratification of human rights conventions, overall finance commitments for respecting human rights, number of employees and community members that have access to complaints, disputes, and grievance processes, access to health insurance). However, the focus on such observable and measurable indicators ignores whether or how the indicators correspond to the ‘actual’ experience of human rights and the ‘real’ properties and mechanisms of human rights. Without attention to the structural features of human rights and social determinants of health, it is difficult to theorize explanatory linkages between them and to develop recommendations that could result in changes to that relationship – and consequential health effects.

From a CR perspective, the way health rights are interpreted and discussed is also based on our understanding that may change over time – they are transitive understandings. The transitive nature can be seen in how legal conceptualisations of the right to health have been broadened over the years. And, if we are to avoid conflating entities with our ideas about them, we need to recognise that rights as ‘real things’ are not the same as our local/personal/temporal interpretations of them.

### Theorising an explanatory framework

**Fig. 1:**
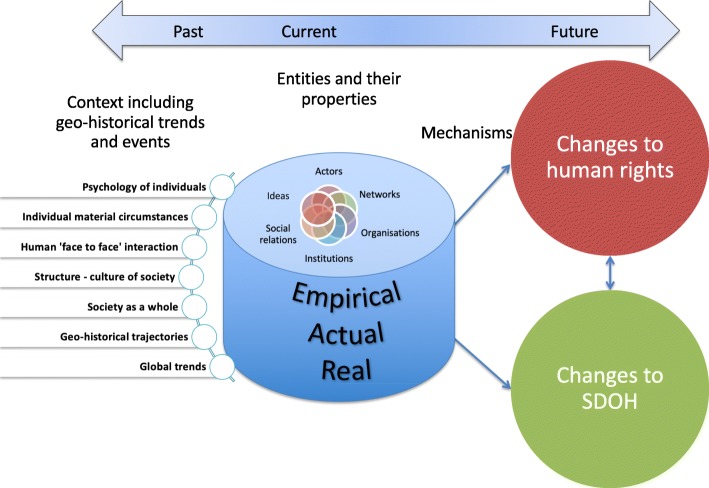
Critical Realist Human Rights and Social Determinants of Health Explanatory Framework

We present a critical realist informed framework for describing the environment that incorporates human rights and social determinants of health-related entities – and defines their relationship (Fig. 1). This framework emphasizes that these entities and relationships can be understood to exist within a stratified, laminated, emergent, open system that contains an assemblage of entities that have a relationship to human rights. Entities in health rights environments can take different forms such as physical, cultural, biological or social. Actors can be described in terms of the social relations and institutional structures they belong to. Actors belong to, and are influenced by, multiple institutions and structural relations – but also have agency to influence and change those structures. Differentiating between actors and structures emphasises people and their capabilities as one unit of analysis and institutions and social relations associated with systems as another. In this context, the key human rights relational structure is that between rights holders and duty bearers.

**Fig. 2:**
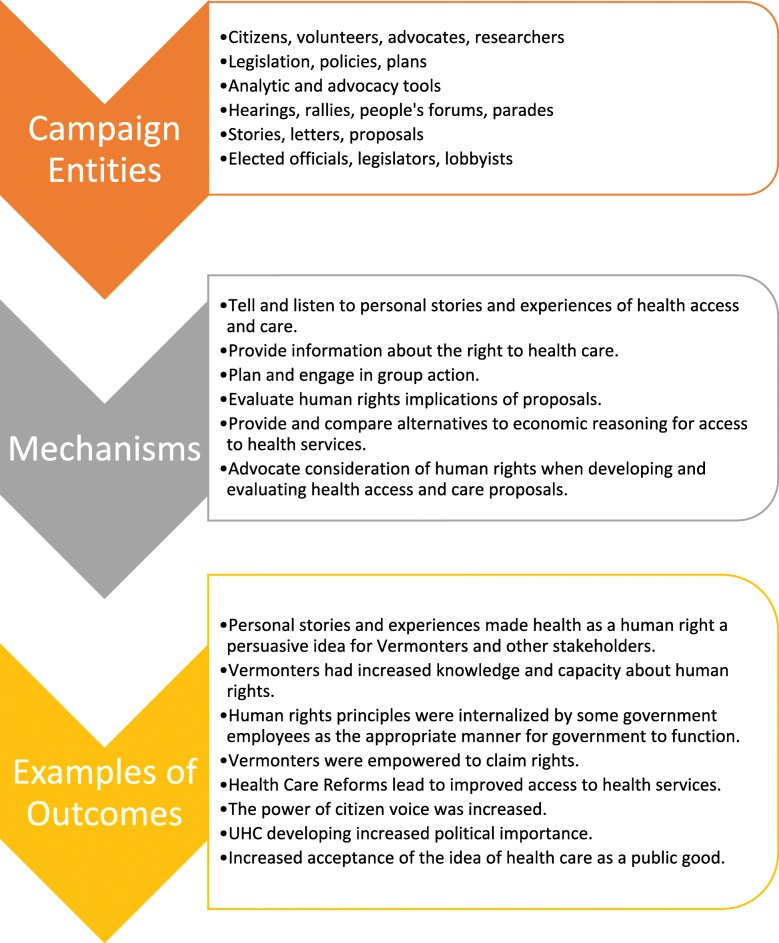
Vermont Case Study: Towards a theory of how the campaign worked

The framework can be subject to substantial elaboration, as below, which emphasizes the complexity of this environment. That complexity is reflected in the array of relationships that potentially exist between the numerous entities involved. Those relationships, which are defined by the activation and effects of mechanisms, explain how the environment ‘works’ (e.g. see Fig. 2). The framework can assist researchers to identify the mechanisms that may be in play and that should be subject to further in-depth investigation and development of explanatory theory. Key features of the framework are now identified and discussed. Some of the potential relationships and associated mechanisms are illustrated using the Vermont case study.

When we conceptualise the spaces where human rights play out as being laminated, we can begin to identify what entities and related mechanisms exist at different laminations and also to consider how the interplay of mechanisms and the specific context influences those mechanisms. Analysis of the relationship between human rights and health that doesn’t take account of the linkages between laminations may result in a focus on specific levels. For example, individual lifestyle factors (such as excessive alcohol use) may be attended to without a concurrent focus on possible more distal causes (for example, the colonisation history and racism within the country) that emanate from other laminations [9, 32]. Bhaskar [33] identifies seven laminations and in the table below we identify examples of HR and SDOH entities and relationships across these laminations (see Table 1). The levels identify people, the physical environment and social structures as key entities. People themselves are also layered and “can be understood as a uniquely laminated layered structure, shaped by genetics, nurture and culture, so that each person has strong and partly predictable tendencies” [34]. People interact with entities and structures across these layers.

**Table 1:** Laminated human rights environment with examples from Vermont Case Study

BHASKAR’S SEVEN LAMINATIONS		EXAMPLES FROM VERMONT CASE STUDY
Psychology of the individual	Internalised norms, values, beliefs, interests, desires, sense of self, reflexive thought, habits. Rights exist within our understanding and knowledge of them [40].	On a personal level many decision makers were supportive of the idea of Universal Health Care. *“I saw people die, young people die of preventable diseases, and it all resulted from them not having health insurance. So for me, it was because I look at this as a human right.”*
Individual material circumstances	The individual or biographical level, experiences, skills, income, education, occupation, level of human rights/SDOH knowledge, health literacy, experience of human rights fulfilment or absence, individual lifestyle factors. The embodied nature of human rights exercise or violation of rights.	In Vermont approximately 66,000 people (11%) have no health insurance at all; this includes more than 11,000 children. *“.*. *. the committees heard so many horror stories about*. *.*. *the way our current health care system treats people kind of like they’re meat and people driving up huge health care bills, going into bankruptcy because of*. *.*. *nothing to do with it, maybe just genetics or an accident. So those were incredibly compelling stories.”*
Human face to face interaction	Interpersonal rights involve respecting the others personal space, time and integrity. Exercise of power - creative, emancipatory and transformative vs destructive, coercive and oppressive [36]. Individuals or small group interactions with stakeholders, decision makers and, communities, interested actors.	The campaign utilised personal stories about how the current health system impacts on lives. “*the ‘Health Care is a Human Right’ campaign really did a great job of getting out and neighbor to neighbor talking to people about health care … And that kind of peer-to-peer outreach I think was helpful in getting kind of the grassroots support.”*
Structures and culture	Human rights culture, structural relations, social class, institutions (rules, conventions, norms, values and customs), ideas (human rights, neo-liberalism, social determinants of health, the value of different types of evidence), policy-making frameworks, decision-making processes.	Vermont is a progressive state with a record of being a leader on human rights issues. Vermont is a relatively small state with a very open and accessible legislature. Legislators became aware of community members demands for UHC- leading to UHC developing increased political importance. *“there’s sort of a direct line from international law and how the right to health has been interpreted down to the Vermont Workers’ Centre framework, down to the law, to the statute in Act 48.”* *“We Vermonters believe that we should take care of our poor and our sick. It doesn’t make financial sense for an insurance company to skim off, you know the easy people to cover and leave the poorest and the sickest for us to cover as a community.”*
Society as a whole	Level of enjoyment/fulfilment of human rights/SDOH, the economy, governance, policies, inequalities, politics, laws, level of development, physical environment and resources, access to services, resources.	There were multiple activities beyond the campaign that were supportive of UHC. Health care policy discourse was influenced by the economic recession. *“One interesting thing that human rights also does is it allows us to become close allies with people who are interested in other human rights issues, whether its unions or migrants rights issues or whatever and it enhances our people power incredibly.”*
Geo-historical trajectories	Traditions, colonisation history, past decisions, morphogenic mechanisms, experiences of actors involved, history of activism and resistance. Rights exist in social structures and history such as colonisation histories [36]. Historical repertoires of power [41]	Vermont has a long history of action around UHC. *“Vermont has been a leader around establishing what some of us called civil rights for same sex couples, legal recognition, and so I think Vermont has been a fertile ground for the concept of health care as a human right also because Vermont has been a leader in establishing and raising the expectations around other types of important issues such as legal recognition.”*
Global trends	Global patterns of inequality, transnational organisations, international human rights agreements and rules, climate change, migration, resource distribution, international social actors, international organisations, International civil society organisations	Health care costs is recognised as a serious and increasing problem. *“I consulted around the world on health care for children. And that was Ireland, Northern Ireland, England, Scotland, Norway, France Israel, Australia, Chile. So I really got a chance to see, in all those countries that every one of them had universal health care and in every one of them, the price tag was considerably short. And in almost every one of them the quality of their health care was quite remarkable. “*

The relationships that exist between entities within and across laminations can often be characterized in terms of the relative power that entities have. Bhaskar describes two types of power relations linked to structure and agency [35]. Human rights infringements are often the result of repressive power relationships that enable some agents to maintain destructive, coercive and oppressive advantages over others’ interests [36]. These power relationships are often related to structures and beliefs related to class, gender, age and ethnicity. At the same time, power relationships can trigger creative, emancipatory and transformative mechanisms that enable and empower agents [36]. Although described by Alderson as different dimensions, these contrastive types of power could also be viewed as the extremes of one dimension (interpersonal relations). We can take account of dimensions of power when developing causal explanations and identifying what to do. In line with CRs emancipatory values, actions should target development of enabling and empowering relationships. Such relationships were evident in the campaign in Vermont which involved civil society actions intended to minimize coercive repressive relationships that were associated with neoliberal health care policies. The latter involved a relationship between access to money and access to health services. Attention to human/health rights emphasizes the need to consider power-related relationships and associated accountabilities, in particular between states and communities. As London and Schneider observe, this can help ensure there is

“the space for civil society action to engage with the legislature to hold public officials accountable and confirms the importance of rights as enabling civil society mobilization, reinforcing community agency to advance health rights for poor communities” [37]

Different types of data and disciplinary perspectives may be required to describe the entities that make up different slices or laminations of reality and the interplay between them [11]. To facilitate understanding of complex health rights environments and decisions about evidence, researchers and practitioners are likely to need to make use of more varied conceptual frameworks that are grounded in different disciplines and their related methodologies [20]. Understanding the role of entities within these different laminations may also require transdisciplinary work that goes beyond disciplines working in parallel or sequence, in order to utilise integrative approaches [38, 39]. CR provides a coherent rationale for, and guidance on, the use of multiple data, methodologies and methods within SDOH and HR research. The coherence rests on the ontological and epistemological perspectives of CR which leads to a pluralist, as well as pragmatic, stance on these considerations.

In Vermont the laminated nature of the relationship between the human rights driven campaign and access to health care is illustrated using examples in Table 1.

As illustrated in Fig. 2, a wide range of mechanisms associated with the varied entities involved in the campaign were activated. These mechanisms related to learning about the right to health, community mobilisation, awareness raising in decision makers, framing of ideas, and responding to new developments. These mechanisms were contingent on contextual factors such as Vermont’s history of being a progressive state and the Vermont Workers Centre being well established with an existing base and relationships. Ultimately the campaign contributed to a number of outcomes described in Fig. 2 including human rights principles being incorporated into Vermont legislation.

## Conclusion

We have argued that in order to advance our knowledge and understanding across a field that is characterised by multiple disciplinary perspectives and approaches, we need to think about the meaning of knowledge and knowing: we need to consider our research paradigm. To confirm this stance, we have presented and account of, and commentary on, our application of the critical realist paradigm in a project focusing on the relationship between HR and SDOH. The presentation is also intended to provide a transferable case study and model of critical realism ‘in action’. While this paradigm now underpins the research of an increasing number of researchers involved in health and rights related research, for many it is unfamiliar, challenging or even troublesome newcomer.

Given this agenda, we have highlighted the following aspects of the CR paradigm:

- Critical realist ontology acknowledges the complexity inherent in social phenomena and provides a conceptual framework for describing this complexity. Descriptions of complexity, as we have illustrated, necessarily go beyond the empirical domain of reality (i.e. beyond what can be observed, experienced and measured).
- Critical realists take a pluralist and pragmatic stance with respect to methodologies and methods that might be drawn on to theorising this complexity - and to the associated use of perspectives and approaches that may be multi-disciplinary, interdisciplinary and transdisciplinary. Critical realists seek to avoid being trapped within the silos of single disciplinary views. When theories that are founded in different paradigm positions and across different disciplines are drawn on, they are re-interpreted through a critical realist ontological lens. This represents a form of ‘abductive reasoning’ which, along with retroduction, is a distinctive feature of a CR theorising methodology.
- CR adopts ‘practical adequacy’ as one of the criteria for evaluating new theory. Does the explanatory theory provide a foundation for actions that can be demonstrated to be beneficial rather than harmful? With this in mind, CR axiology supports social critique as a dimension of the research process.
- Critical realists recognize that the constancy of change and emergence means that a ‘settled’ theory concerning the relationships between phenomena cannot be formulated. This calls into question the notion of determinants, as the term can imply a degree of stability that is not present. Constructs and propositions may be transient. At the same time, some differentiation of entities (properties, mechanisms and relationships) that may be relatively stable is possible, as illustrated in the Vermont case study.

The key features of human rights and SDOH environments, identified as an our outcome of our theorising work, include the following:

- HR and SDOH environments are understood to be open, laminated, complex and adaptive systems.
- Entities can take different forms such as physical, cultural, biological or social.
- Actors can be described in terms of the social relations and institutional structures they belong to.
- There is intersectionality of actors whereby actors belong to, and are influenced by, multiple institutions and structural relations - and can also be simultaneously individual, primary and corporate actors.
- Understanding and explaining the relationship between human rights and SDOH requires going beyond the observable to consider structures, powers, and mechanisms and requires transdisciplinary work.

With respect to practical implications of our theorising work, we argue that successful implementation of global initiatives such as the Sustainable Development Goals requires more than the setting of targets and indicators. Structural analysis and development of explanatory theory is necessary if we are to understand what things are, how they work – and how they might work better. This type of research will enable the fields of public health and human rights to identify the fundamental causes of health and human rights inequities such as economic structures, class and racism, and to conceive ways of addressing them. Explicit and indepth consideration of the relationship between human rights and the social determinants of health is critical to strengthening accountability and governance mechanisms.

Finally, we recommend some practical steps to facilitate greater consideration of the place of paradigms in research on human rights and social determinants of health. As researchers when reporting on research on SDOH and HR, we can outline, as in this paper, the paradigm perspectives that influenced our research and related assumptions about the knowledge that we have constructed and evaluated. As practitioners, we can have conversations in our work with communities and other stakeholders about how we understand knowledge, the role of different types of evidence and ways of theorizing explanations and evaluating their practical adequacy. We cannot and should not assume that our views about these matters are shared by others. However, as Huber and Morreale [42] observe about interdisciplinary encounters

growth in knowledge also comes at the borders of disciplinary imagination....It is in this borderland that scholars from different disciplinary cultures come to trade their wares – insights, ideas and findings – even though the meanings and methods behind them may vary considerably (p. 1)*.*

## Data Availability

N/A

## Abbreviations

CR: Critical realism
HIA: Health Impact Assessment
HR: Human rights
SDOH: Social Determinants of Health
UHC: Universal Health Care
UN: United Nations
VWC: Vermont Workers’ Centre
WHO: World Health Organisation
